# Chit-Chat

**Published:** 1896-02

**Authors:** 


Secretary.

---

### CHIT-CHAT.

The Ætna Life Insurance Company of Pennsylvania, with headquarters at Philadelphia, and which recently passed into the hands of a receiver, almost daily conducts suits against former stockholders for unpaid assessments. With but one exception so far, this has been the history and end of every live-stock insurance company organized in Pennsylvania.

One-third of the graduates failed at the December examination of the Pennsylvania State Medical Board.

William J. Waugh, V. S., Third Cavalry, U. S. Army, was a recent visitor, as a court-martial witness, at Ft. Leavenworth, Kan., where a medical officer was being tried by a general court-martial on charges preferred by a fellow-officer of the Medical Department of the U. S. Army.

William Nicholson, V. S., a recent graduate of the Ontario Veterinary College, has located in Allegheny, Pa., and bids for public patronage by billing the town with yellow placards 14 x 22 inches, with very black letters.

Dr. Leonard Pearson, consulting meat inspector to the Department of Public Safety in Philadelphia, has tendered his resignation, to take effect on the appointment of his successor.

Pittsburg is worked up over the death of a large number of elk in Schenley Park, as upon whom they shall lay the blame. From bad sanitary conditions, it appears the elk became infested with parasites, and lack of attention and improper food are said to have contributed to the great fatality among them.

A school of anatomy and scientific horseshoeing has been established in Chicago, under the direction of the Master Horseshoers' Association. Prof. Hughes, of the Chicago Veterinary College, opened the course by a lecture on the anatomy of the horse, which composed one of the series which he will deliver. Dr. Frank Allen delivered the second lecture of the course.

The *Humane Advocate* enters the field of journalism with the incoming new year, at Pittsburg, Pa. It is well printed, and will endeavor to serve for the people of that section what similar journals are doing in other cities. It has identified with it, as veterinarians, Drs. E. J. Carter and James A. Waugh. The first number contains articles from the pens of Veterinarians Hancock and Waugh. Editorially, the *Advocate* will be directed by Misses Kyle and Docking.

The New York College of Veterinary Surgeons reports a very nice case of filaria oculi, which will be operated upon after proper preparation. It was sent to the college by Dr. P. C. Hogg, Manhasset, L. I.

A recent issue of the *Pittsburg Medical Review* contains a very interesting article from the pen of Veterinarian J. Stewart Lacock, V. M. D.; Allegheny, Pa., describing a post-mortem on a rabid canine, with notes on inoculation of virus into rabbits, in Pittsburg Biological Laboratory, in connection with the Bureau of Health.

Dr. C. A. Cary, as Veterinarian to the Alabama Agricultural Experiment Station, has just issued Bulletin No. 67, on the subject of tuberculosis. He very thoroughly covers the ground in the interest of the people and live-stock owners of his State, and we hope and believe that it will lead to control measures of importance and great value to all concerned.

Pittsburg has just accepted a gift of one hundred thousand dollars from Mr. Christopher Magee, one of her warm and generous-spirited citizens, to be used in establishing a Zoölogical Garden in Highland Park, the latter overlooks the Allegheny River and Valley, which makes it a very suitable location for such a purpose.

Experiments are being made with the germ recently cultivated of distemper in dogs.

Prof. Adami has recently solved the cause of the obscure disease familiarly known to veterinary readers as the "Pictou Cattle Disease." It is of germ origin.

Truth is a virtue, but a mighty awkward one to handle in a horse trade.

A libel suit is under way involving several veterinarians, who have expressed opinions as to the soundness of a pair of horses exhibited at both the New York and Philadelphia shows, and which were sent from the show-ring as unsound.

A suit for damages resulting from the feeding of Indian peas, among a stable of horses in Birmingham, England, has been decided in favor of the plaintiff.

The horse is filling still another grand field at the Pasteur Institute, Paris, in serving for the preparation of another serum, for combating the streptococcus microbe, the now considered agent of production of erysipelas, puerperal fever, etc.

The so-called "corn-stalk disease" has been quite prevalent throughout certain districts in Iowa during November and December.

U. S. V. M. A. (State Secretary's Report): Montana reported five graduates within her borders; a graduate succeeding a non-graduate as State Veterinarian. Only two veterinarians in general practice. No inducements were offered for those wishing to engage in general practice.

The production of tetanus, antitoxin, and mallein has been commenced at the New York College of Veterinary Surgeons.

Argentine threatens to become America's strongest compeitor in the world's markets for supplying animal food-products.

St. Paul and Minneapolis will adopt rules and regulations governing inspection of dairies supplying the twin cities with milk, similar to those in force in Winnipeg, Manitoba.

Kansas City, Mo., is already in the field for the 1897 meeting of the United States Veterinarian Medical Association.

The New York Veterinary College Laboratory is producing diphtheria antitoxin of over 3000 units. This, in the director's opinion, is due to improvements in the process of producing the toxin, which is made at the laboratory of the New York Health Department under the direct supervision of Dr. Herman M. Biggs. A year ago the strongest, according to the foreign process they could get, was $\frac{1}{20}$th strength, to-day they are using $\frac{1}{200}$th strength, which is ten times as strong, and makes a very considerable difference in the quantity injected, and seems to produce very much stronger antitoxin.

